# Improved gliotransmission by increasing intracellular Ca^2+^ via TRPV1 on multi-walled carbon nanotube platforms

**DOI:** 10.1186/s12951-022-01551-1

**Published:** 2022-08-11

**Authors:** Won-Seok Lee, Ji-Hye Kang, Jung-Hwan Lee, Yoo Sung Kim, Jongmin Joseph Kim, Han-Sem Kim, Hae-Won Kim, Ueon Sang Shin, Bo-Eun Yoon

**Affiliations:** 1 Department of Molecular Biology, Dankook University, Cheonan, 31116 Republic of Korea; 2 Department of Nanobiomedical Science, BK21 FOUR NBM Global Research Center for Regenerative Medicine, Dankook University, Cheonan, 31116 Republic of Korea; 3 Institute of Tissue Regeneration Engineering (ITREN), Dankook University, Cheonan, 31116 Republic of Korea; 4 Department of Biomaterials Science, College of Dentistry, Dankook University, Cheonan, 31116 Republic of Korea; 5 Dental Medicine Innovation Centre, UCL Eastman-Korea, Dankook University, Cheonan, 31116 Republic of Korea; 6 Mechanobiology Dental Medicine Research Center, Cheonan, 31116 Republic of Korea

**Keywords:** Astrocyte, Carbon nanotubes, Glia, Gliotransmission

## Abstract

**Background:**

Astrocyte is a key regulator of neuronal activity and excitatory/inhibitory balance via gliotransmission. Recently, gliotransmission has been identified as a novel target for neurological diseases. However, using the properties of nanomaterials to modulate gliotransmission has not been uncovered.

**Results:**

We prepared non-invasive CNT platforms for cells with different nanotopography and properties such as hydrophilicity and conductivity. Using CNT platforms, we investigated the effect of CNT on astrocyte functions participating in synaptic transmission by releasing gliotransmitters. Astrocytes on CNT platforms showed improved cell adhesion and proliferation with upregulated integrin and GFAP expression. In addition, intracellular GABA and glutamate in astrocytes were augmented on CNT platforms. We also demonstrated that gliotransmitters in brain slices were increased by ex vivo incubation with CNT. Additionally, intracellular resting Ca^2+^ level, which is important for gliotransmission, was also increased via TRPV1 on CNT platforms.

**Conclusion:**

CNT can improve astrocyte function including adhesion, proliferation and gliotransmission by increasing resting Ca^2+^ level. Therefore, our study suggests that CNT would be utilized as a new therapeutic platform for central nervous system diseases by modulating gliotransmission.

**Graphical Abstract:**

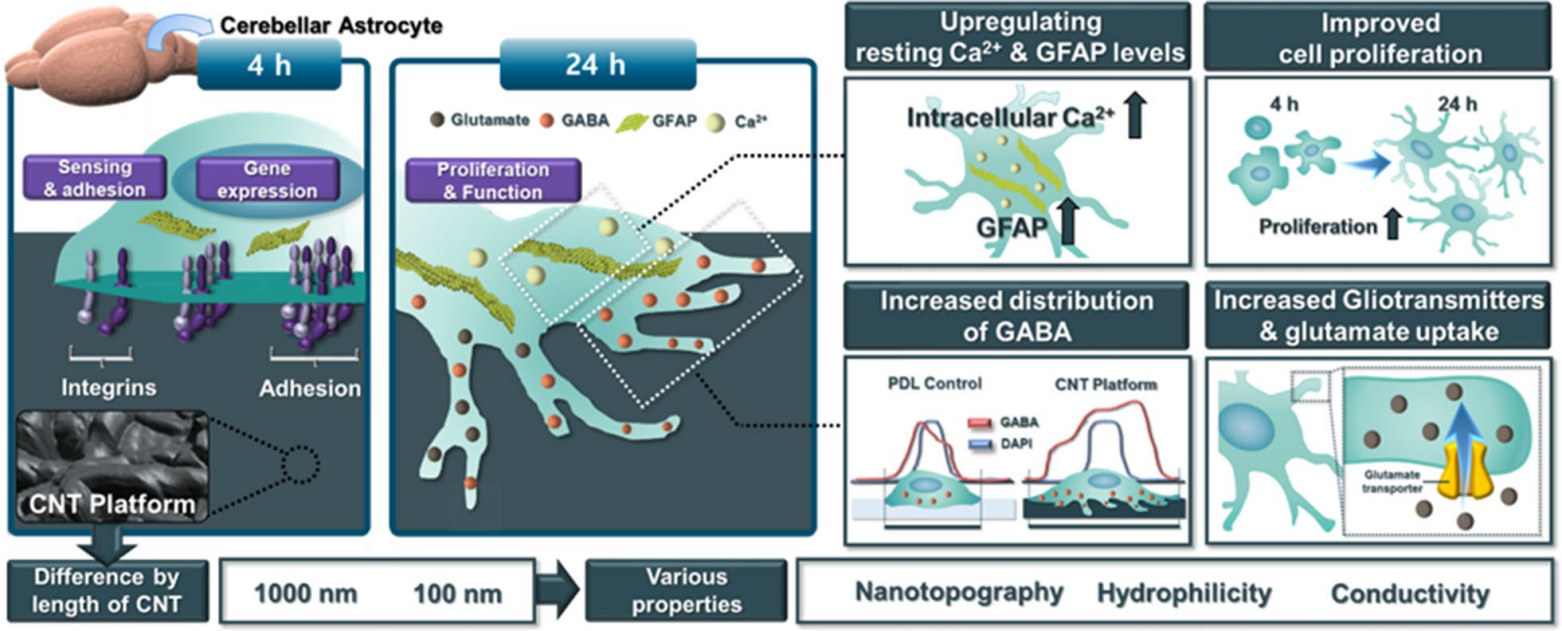

**Supplementary Information:**

The online version contains supplementary material available at 10.1186/s12951-022-01551-1.

## Background

Astrocytes, the most abundant cell among the glial cell types, directly contact neurons by forming a tripartite synapse and have diverse receptors for the corresponding neurotransmitters and neuroactive molecules like neurons [1–3]. They also modulate neuronal activity by releasing gliotransmitters [4–6]. Recently, astrocytes have been implicated in neurodegeneration, such as Alzheimer’s disease (AD) and Parkinson’s disease (PD) [7, 8]. Growing evidence has revealed that reduced gliotransmitters from astrocytes, such as ATP and glutamate, induce tau-mediated synaptic dysfunction [9], and that abnormally released astrocytic gamma-aminobutyric acid (GABA) can provoke the memory impairment in an AD mouse model [10].

Among gliotransmitters, GABA and glutamate are metabolically interconnected and are closely linked to intermediate metabolism [11]. These amino acids are synthesized and released by neurons and astrocytes [12–15]. GABA mediates tonic inhibition in Bergmann glial cells and lamellar astrocytes. Tonic inhibition was first identified as particularly prominent in the cerebellum and can occur because of the resting level of calcium ion (Ca^2+^) in astrocytes [12, 13]. Intracellular Ca^2+^ in astrocytes is essential for interaction with neurons and has a function as a counterpart of neuronal membrane potential change. In addition, these intracellular Ca^2+^ signals can induce the release of gliotransmitters from astrocytes [16–18]. In turn, astrocytes can participate in the tripartite synapse by modulating the excitation/inhibition (E/I) balance, which is critical for neuronal activity and central nervous system (CNS) homeostasis [19, 20]. For instance, astrocytes can control motor coordination by modulating E/I balance via gliotransmission in the cerebellum [21]. Therefore, regulation of gliotransmission may be a feasible therapeutic target for neurodegenerative diseases.

The effects of diverse nanomaterials on glial cells have been studied recently, including polymeric, metallic, metal oxide, and carbon-based nanomaterials such as carbon nanotubes (CNT) and graphene oxide, and their functionalization [22]. However, most nanomaterials have been used to penetrate astrocytes by spiking the suspension in vitro*.* Penetration into cells has drawbacks that include cytotoxicity (apoptosis and necrosis), damage to cytosol compartments (lysosome, endosome, cytosol, mitochondria), degradation of materials, and failure to maintain the structure until it affects cellular function [23]. To avoid these drawbacks, cells must be exposed to an environment where nano-topography and chemical properties of the material are applied [24]. However, the effect of astrocyte exposure to these microenvironments has not yet been studied.

Nano-topography and the chemical properties of materials depend on many factors that can modulate the physiological behavior of cells to perform their function [24]. For instance, increasing the porosity of nanoporous gold reportedly did not affect cell density, but induced a decrease in astrocyte area, which suggests that nano-topography can adjust to modulate cellular adhesion [25]. In another study, electrospun fibers made of poly L-lactic acid (PLLA) affected cortical astrocytes; the latter were significantly shorter and broader on shallow grooved and small indented fibers compared to those on smooth fibers, indicating that astrocytes respond differently to the presence of nano-topography [26]. However, most of these studies have focused on cell adhesion and morphology. Few studies have identified glial cell functions, such as gliotransmission or intracellular Ca^2+^ changes.

In our previous study, the CNT platform increased cell-to-cell interactions in cortical astrocytes, which was confirmed by observing the levels of glial fibrillary acidic protein (GFAP) and GABA distribution in the cell process [3]. In this study, we fabricated CNT platforms to provide a non-invasive microenvironment for cells with different nano-topographies and properties such as hydrophilicity and conductivity. We identified adhesion and subsequent proliferation that occur when cells interact with nanomaterials. Furthermore, we confirmed through in vitro, and ex vivo experiments whether CNT could regulate gliotransmission and glutamate uptake ability according to the intracellular resting Ca^2+^ level of astrocytes. This study suggests that CNT can improve astrocytic proliferation and gliotransmission-related functions and can be used as a modulating nanomaterial for neuron-glia interaction.

## Results and discussion

### Preparation of functionalized multi-walled carbon nanotubes (MW-CNTs)

**Fig. 1:**
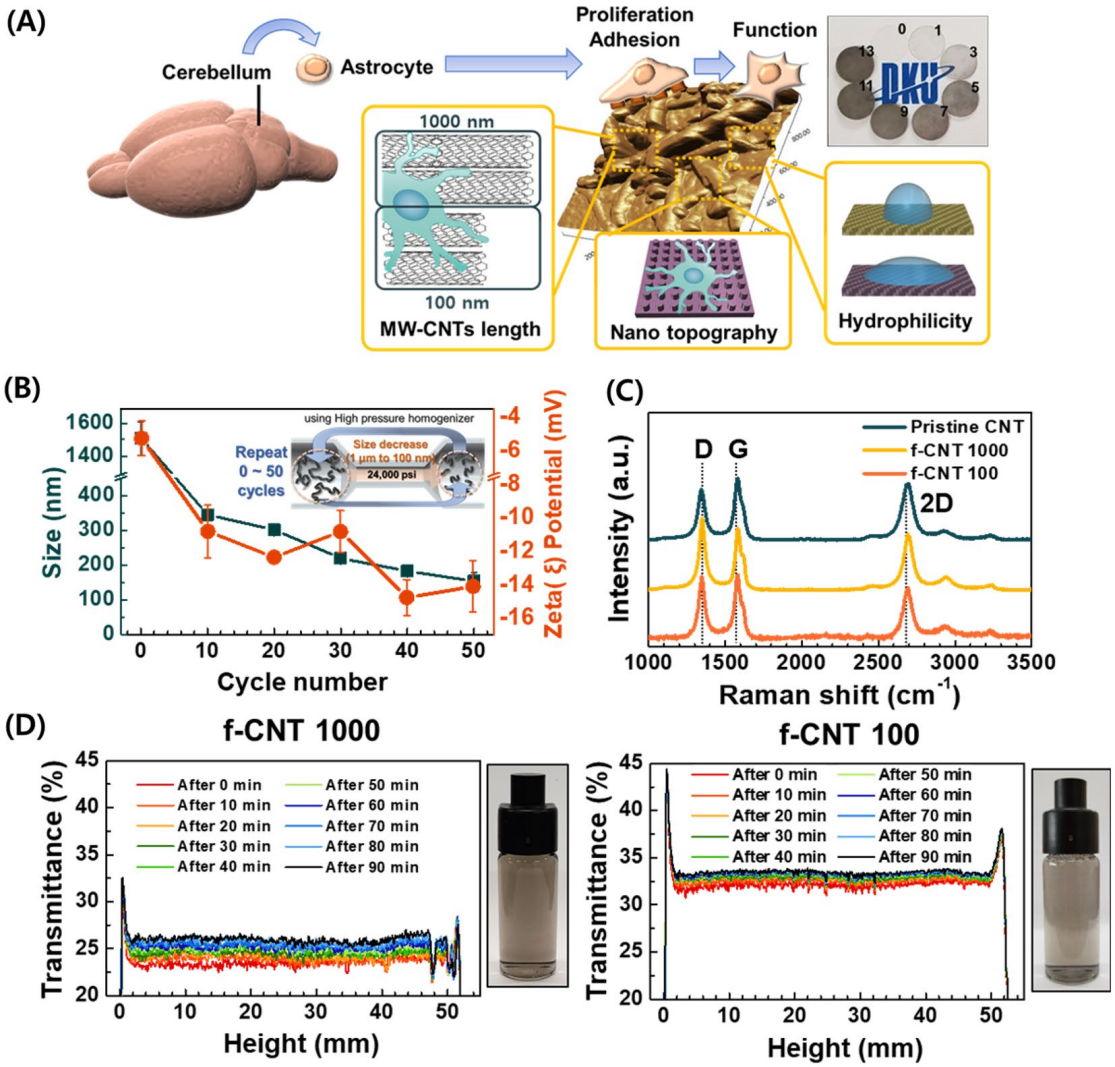
Characterization of functionalized carbon nanotubes (CNTs) to synthesize the CNT platform. **A** Schematic workflow diagram of CNT platform for interaction with astrocyte and identifying its properties. Determination of cell viability, functionality, and gliotransmitter levels in cerebellar astrocytes. Adjustment of functionalized CNT length to nano-topography formation and various hydrophilicity. The insert photograph in the upper right shows stack of coated glasses by overlapping degrees. **B** Size and zeta (ξ) potential of functionalized CNTs. **C** RAMAN spectra of pristine CNT, f-CNT 1000, and f-CNT 100. **D** Dispersion stability of f-CNT 1000 and f-CNT 100 for 90 min

We designed the CNT platform with various properties and observed the viability and functionality, including proliferation and gliotransmission of cerebellar astrocyte in mouse brain (Fig. 1A). Chemically coating modified MW-CNTs prepared the platform by length on glass, which induced nano-topography with their roughness. In addition, the hydrophilic level of the platform was different and subsequent adhesion, spreading and function of astrocytes were identified on the various topography and hydrophilicity.

To prepare the MW-CNT platform with various properties, it was chemically and physically functionalized. For chemical functionalization, chemical cutting (average 1000 nm) adjusted the MW-CNT length and oxidized the MW-CNTs (Step1), and the MW-CNTs were physically cut repeatedly using the high-pressure homogenizer for physical functionalization (Step2) (Fig. 1B, Insert). Therefore, a cycle of 50 repetitions reduced the CNT length from 1510.3 nm to 153.7 nm (Fig. 1B, left y-axis). The chemical oxidation of the edge and physical cutting increased the absolute value of zeta potential in the negative direction (Fig. 1B, right y-axis). This result indicated that water solubility increased by reducing the MW-CNT size after functionalization. We selected f-CNT 1000 (about 1000 nm long) as the representative model for chemical functionalization (Step1) and cut it for 50 cycles to prepare f-CNT 100 (about 100 nm long), the representative model for chemical and physical functionalization (Step2).

The typical RAMAN spectra analysis of the functionalized CNTs (f-CNT 1000, f-CNT 100) is shown in Fig. 1C. D band intensity (I_D_) is attributed to crystalline network symmetry, which is present in defective materials. G band intensity (I_G_) suggests the stretching of the *sp*^2^ bond. I_D_/I_G_ ratio can be used to quantify the amount of defect in carbon-based materials [27, 28], which increased in MW-CNTs through functionalization (Table 1). As detailed in RAMAN spectra analysis, the in-plane crystalline size (L_O_) is an indirect measurement through inter-defect distance with the surface [27, 28]. The L_O_ of f-CNT 1000 and f-CNT 100 also increased, which is valid for the disagreement of an average size of *sp*^2^ domains with increasing I_G_/I_D_ ratio [28]. These results indicate that both f-CNT 1000 and f-CNT 100 surface drastically changed after acid treatment and physical process compared to pristine CNT surface.

**Table 1:** Structural parameters of the functionalize MW-CNT samples obtained from RAMAN spectra analysis

Sample	D band intensity (I_D_; cm^−1^)	G band intensity (I_G_; cm^−1^)	I_G_/I_D_ ratio	L_O_ (nm)
Pristine CNT	1344	1582	0.83	15.98
f-CNT 1000	1348	1584	0.98	18.81
f-CNT 100	1349	1585	1.22	23.45

Additionally, Fourier-transform infrared spectroscopy (FTIR) results exhibited the assigned peaks (–OH, –(C = O)–, and –COOH) in Step1, Step2) related to oxidation in MW-CNTs (Additional file 1: Fig. S1). Moreover, thermogravimetric analysis (TGA) results showed that thermal degradation was rapid with respect to the degree of oxidation and size reduction, confirming the results of the RAMAN spectra analysis (Additional file 1: Fig. S1). In addition to the hydrophilic properties, we recorded the transmittance at 37 °C to determine the dispersion and thermal stability (f-CNT 1000 and f-CNT 100 shown representatively in Fig. 1D. Results by cycle are shown in Additional file 1: Fig. S2). The transmittance gap of each sample for 90 min was 3.2% in f-CNT 1000 and 1.8% in f-CNT 100. The aqueous solution of functionalized MW-CNT samples is shown in the inset photos. These results demonstrated the hydrophilicity of modified MW-CNTs, which has undergone both chemical and physical functionalization and is hydrophilic and stable, confirming results consistent with the zeta potential. Particularly, it was highly homogeneous, and nothing was not precipitated at the bottom (height: 2.5–10 mm) even after several hours (Additional file 1: Fig. S2). Consequently, these results indicate that MW-CNT size was adjustable, and hydrophilicity and dispersion stability differed depending on MW-CNT length and oxidation depending on the process.

### Characterization of MW-CNT substrate by length for astrocyte

**Fig. 2:**
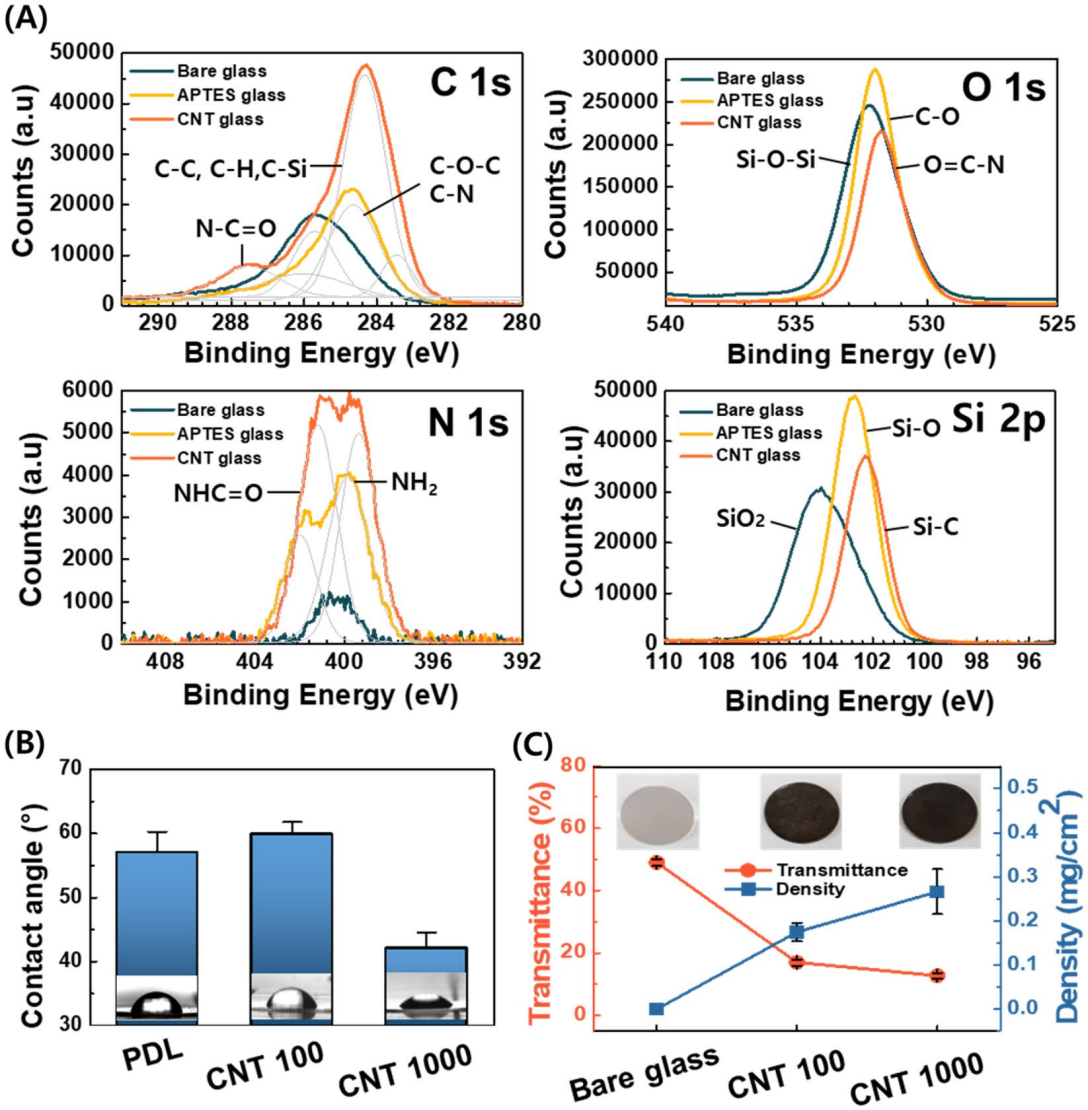
Functional traits on MW-CNT substrates. **A** XPS spectra of the substrate before, during, and after functionalized CNT coating. **B** Contract angle measurements of CNT 100, CNT 1000, and poly-D-lysine (PDL). **C** Transmission and density profiles of the samples with or without coating. The inset photograph shows the photograph of 10 layers overlaid with each sample

To synthesize the platform for interaction with astrocytes, functionalized MW-CNTs of varying lengths were immobilized on the glass substrate with different nano-topography and hydrophilicity. The platform coated with f-CNT 1000 is termed “CNT 1000,” while that coated with f-CNT 100 is termed “CNT 100.” X-ray photoelectron spectroscopy (XPS) analysis was performed on bare glass, intermediate glass (APTES glass), glass coated with f-CNT (CNT glass), all of which revealed the presence of C 1s, O 1s, N 1s, and Si 2p (Fig. 2A, Additional file 1: Fig. S3). The C 1s band of CNT platform was deconvoluted into C backbone assigned peaks as the amount of functionalized CNT sample coated on the glass substrate was increased. Moreover, O 1s, Si 2p band shifted toward low binding energy values pursuant to the coating process on the substrate. Notably, the amide bonds connecting functionalized CNT and glass substrate were simultaneously revealed at 287.4 (C 1s), 530.6 (O 1s), and 400.7 (N 1s) eV, indicating the presence of the amide peak exists in the grafting modified CNT through an amide bond.

While comparing the hydrophilicity with CNT platforms and the controls of each process, we determined that hydrophilicity increased as the oxidation increased with the length reduction (Fig. 2B). Particularly, the hydrophilicity of CNT 1000 was similar to that of poly-D-lysine (PDL)-coated control glass, suggesting CNT 1000 can be used as the materials involving nano-topography patterns under the same hydrophilicity.

**Fig. 3:**
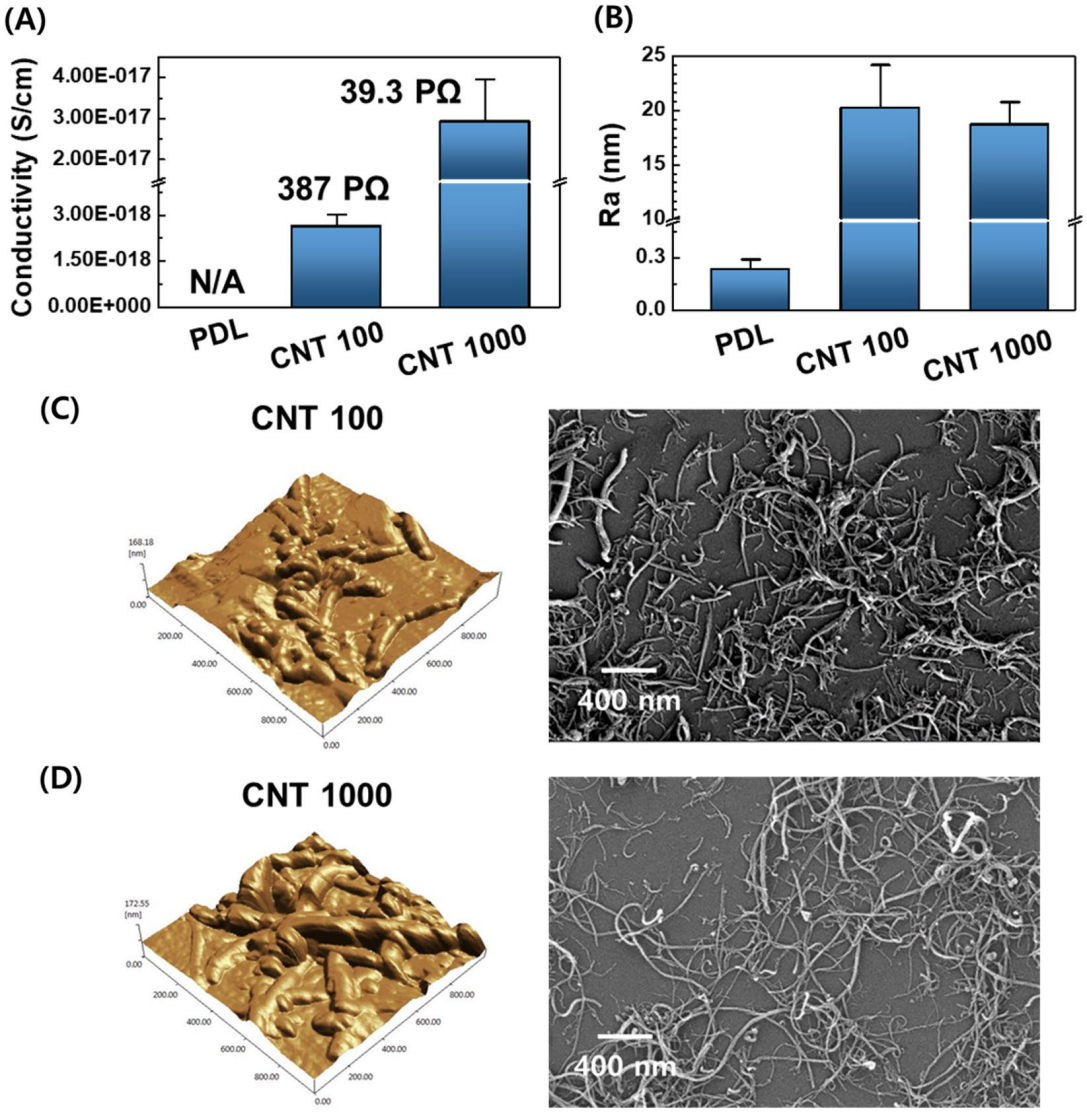
Morphological traits on MW-CNT substrates. **A** Electronic conductivity test for the composites. **B** Average roughness (Ra) of the substrate coated with or without CNT by length. (**C**, **D**) Atomic field microscopy (AFM) topographic images and field emission scanning electron microscopy (FE-SEM) images showing the morphological characteristics of **C** CNT 100 and **D** CNT 1000

The results are shown in Fig. 2C indicates the variations in the sample transmittance and density profiles with or without functionalized MW-CNT coating. The CNT platforms were transparent throughout the coating process, which is comparable with bare glass. Still, when each glass was overlaid with 10 CNT layers (inserted photograph in Fig. 2C), the presence of CNT created a visible difference (Additional file 1: Fig. S4). In addition, the density of platforms was 0.17 mg/cm^2^ for CNT 100 and 0.27 mg/cm^2^ for CNT 1000, which affected overall conductivity, suggesting that CNT 1000 was 10 times better conductive than CNT 100 (Fig. 3A).

The MW-CNT substrates exhibited highly roughened nano-topography based on atomic field microscopy (AFM) and field emission scanning electron microscopy (FE-SEM; Fig. 3C–D). The average roughness (Ra) of CNT 100 (20.2 ± 3.9 nm) is analogous to that of CNT 1000 (18.9 ± 2.1 nm), which was dependent upon MW-CNT fiber diameter (below 20 nm). In particular, the nano-roughness of the CNT platforms (19.5 nm) was 65 times higher than that of the PDL-coated control (0.3 nm, Fig. 3B). Additionally, AFM and FE-SEM images indicated a clear morphological change in the homogenously dispersed nanotubes (Right side of Fig. 3C and D, Additional file 1: Figs. S5 and S6). This roughness of CNT platforms indicated the oxidative damage to MW-CNT surface and morphological change throughout the length, implying the possible effect on astrocyte functions.

### CNT platforms upregulate integrin expression to improve cell adhesion and proliferation

**Fig. 4:**
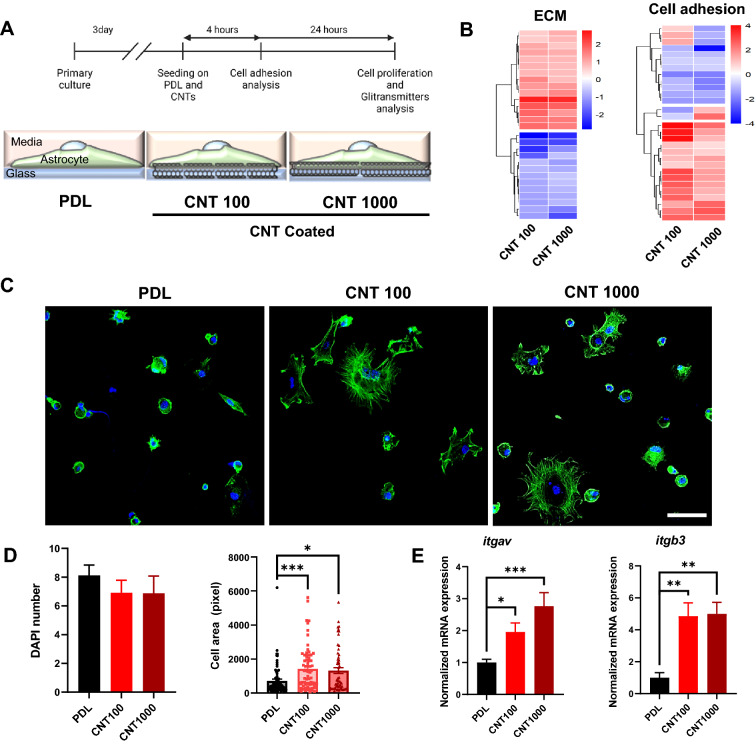
Cell adhesion in astrocytes on MW-CNTs. **A** Schematic diagram of experiments. **B** Heat map illustrating 3′ mRNA seq of ECM and cell adhesion associated genes of primary astrocytes. Illustrating with reference to GO:0031012 (ECM) and GO:0007155 (cell adhesion). **C** Cerebellar astrocytes were examined using confocal microscopy and immunofluorescence with DAPI (blue) and phalloidin (green) after 4 h seeding. Scale bar, 50 μm. **D** The DAPI counts are identical for each group and the cell area on CNT platforms is larger than PDL. **E** The mRNA levels of astrocytes on CNT platforms were normalized to that of the control group and appeared as fold changes. Integrin mRNA levels (ItgaV and Itgb3) were significantly upregulated on CNT platforms (n = 4 from three independent cell preparations)

**Fig. 5:**
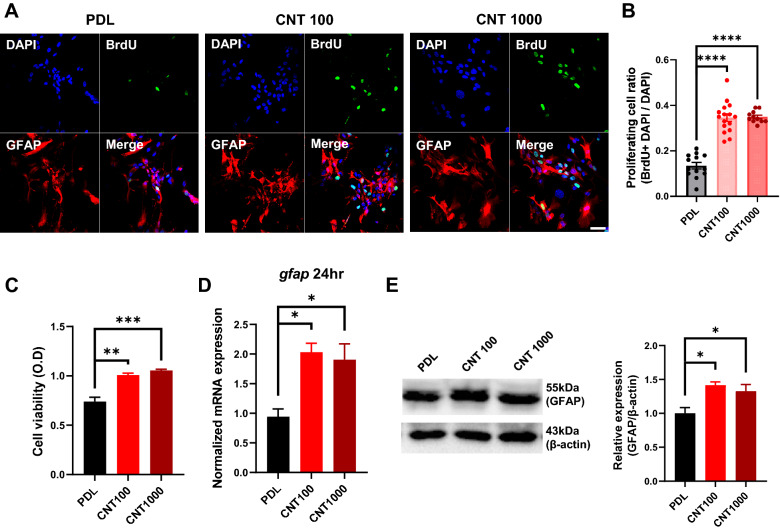
Cell proliferation and GFAP in astrocytes on MW-CNTs. **A** Cerebellar astrocyte cultured on PDL and CNT platforms 24 h after seeding were examined using confocal microscopy and immunofluorescence with DAPI (blue), anti-BrdU antibodies (green) and anti-GFAP antibodies (red). Scale bar, 50 μm. **B** The graph shows that astrocytes cultured on CNT platforms have more BrdU-positive DAPI spots than PDL (n = 45 cells). **C** The graph shows astrocytes 24 h after seeding on PDL and CNT platforms. There are more viable cells on CNT platforms than PDL. **D** The represented mRNA levels of astrocytes on CNT platforms were normalized to that of the control group and appeared as fold changes. In primary astrocytes on CNT platforms, *gfap* mRNA levels were significantly upregulated in 24 h than PDL (n = 4 from three independent cell preparations). **E** GFAP protein levels in cerebellar astrocytes significantly increased, as shown using western blotting analysis (n = 3 from three independent cell preparations)

To assess the effect of CNT platforms, we used primary culture of cerebellar astrocytes from P0-P2 mice. Several analyses were performed to determine any changes in adhesion, proliferation, and gliotransmission of astrocytes 4 and 24 h after seeding on CNT platforms (Fig. 4A). First, we performed Quantseq 3` mRNA sequencing 4 h after seeding. Gene expressions related to extracellular matrix (ECM) and cell adhesion, which is important for initial adhesion [29], were altered on CNT platforms (Fig. 4B). Therefore, we stained astrocyte F-actin with phalloidin to observe the morphological change on CNT platforms 4 h after seeding. Cell area on CNT platforms was larger than poly-D-lysine (PDL)-coated coverslips (Fig. 4C, D). Next, we analyzed gene expression of integrin αV (ItgaV) and β3 (Itgb3) because they are essential for astrocyte–neuron interaction and changes in the actin cytoskeleton [30]. Both integrins were upregulated 4 h after seeding on CNT platforms than PDL (Fig. 4E), implying that the initial adhesion of astrocytes on CNT platforms was enhanced as cell adhesion area and integrin expression are closely related [31]. In addition, we stained yes-associated protein (YAP), which is associated with cell–ECM interaction and regulates focal adhesion [32]. Immunofluorescence analysis of DAPI-positive and cytosolic signal revealed YAP intensity was increased in astrocytes on CNT platforms than on PDL. In contrast, the nucleus/cytosol (N.C) ratio of YAP was not affected (Additional file 1: Fig. S6A, B). These results indicate that overall intensity was increased because astrocytes on CNT platforms have a wider cell area than on PDL. We next investigated whether the improved initial adhesion by CNT platforms affects cell proliferation. Therefore, we stained bromodeoxyuridine (BrdU), a nuclear acid mimetic incorporated into genomic DNA during cell proliferation [33], to confirm whether cell proliferation was improved (Fig. 5A). The number of BrdU-stained nuclei in astrocytes was enriched on CNT platforms, indicating that astrocytes on CNT platforms more proliferated (Fig. 5B). Next, we also performed cell viability assay using cell counting kit-8 (CCK-8). After seeding for 24 h, the viability of astrocytes seeded on CNT platforms was improved than on PDL (Fig. 3G). CNT platforms induce stable adhesion by upregulating integrin gene expression in astrocytes, which is involved in phase II of the adhesion stage [34] and enhances cell stabilization to attach and spread on biomaterials [35]. Nano-topographical differences between PDL and CNT platforms affect the mechano-sensing of astrocytes to recognize and adhere to biomaterials [36]. Due to stable cell adhesion, astrocytes displayed enhanced cell proliferation without cytotoxicity on CNT platforms.

### CNT platforms increase GFAP expression and intracellular gliotransmitters of astrocytes

**Fig. 6:**
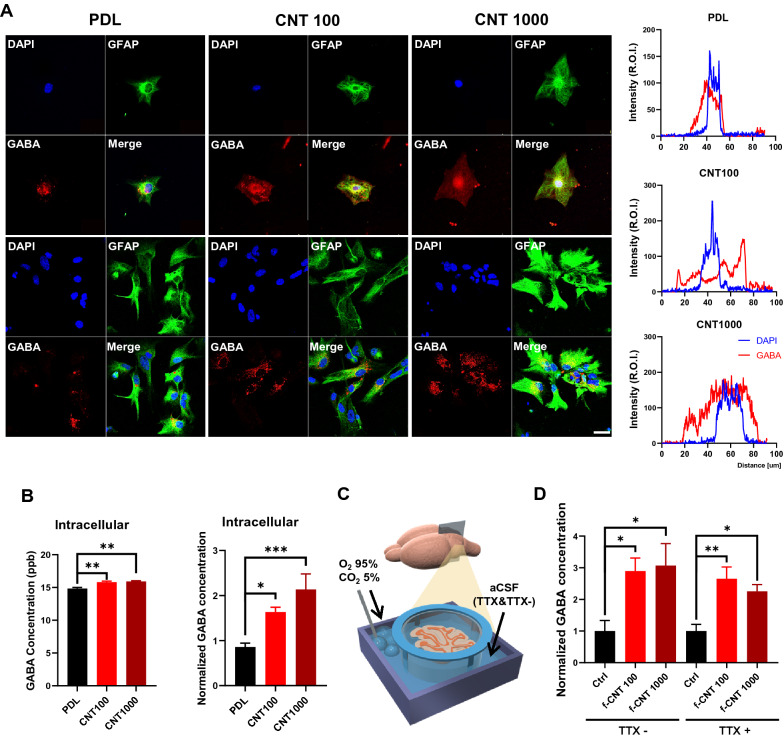
Cellular functions associated with gliotransmission of astrocytes on MW-CNTs. **A** Immunostaining cerebellar astrocytes on PDL and CNT platforms. Scale bar, 20 μm. GABA distribution is more widespread in the cell process direction on CNT platforms. Data are presented as the mean (n = 15 cells). **B** The intracellular GABA was measured using LC/MS and ELISA, which increased on CNT platforms (n = 4). **C** Schematic diagram of cerebellar slices incubated with functionalized CNTs ex vivo. **D** Graphs show that functionalized CNTs increase GABA in cerebellar astrocytes after neurotoxin treatment (TTX) (n = 4 individual mice)

As indicated above, we showed CNT platforms improved astrocyte adhesion and proliferation. GFAP, an astrocyte marker, indicates that astrocyte's status and proliferation can be known depending on expression level [37]. Therefore, we assessed GFAP expression level in astrocytes on CNT platform and PDL. The *gfap* gene expression increased when seeding for 24 h (Fig. 5D). Additionally, we confirmed that GFAP protein expression level increased in astrocytes on CNT platforms by western blot (Fig. 5E). GFAP expression is closely associated with astrocyte function, potentially modulating synaptic function and interaction with neurons [38–40]. Astrocytes sense synaptic activity and release gliotransmitters such as glutamate and GABA that play an important role in neuronal information processing [12, 41, 42]. Therefore, we investigated intracellular GABA distribution in astrocytes on CNT platforms and PDL. Astrocytic GABA was distributed in the cellular process on CNT platforms rather than PDL (Fig. 6A). Next, we used liquid chromatography–mass spectrometry (LC–MS) and enzyme-linked immunosorbent assay (ELISA) to determine whether GABA distribution was due to the altered intracellular levels. Consistently, intracellular GABA level was increased in astrocytes on CNT platforms than PDL (Fig. 6B). We also measured glutamate concentration using glutamate enzyme assay. The total astrocytic glutamate on CNT platforms remains unchanged but extracellular glutamate was increased (Additional file 1: Fig. S8A). These results suggest that CNT platforms upregulate intracellular gliotransmitters in astrocytes, which enhances the chance of gliotransmission. To confirm that these changes of gliotransmitters also can occur in the brain tissue, we incubated cerebellar slices with f-CNT 100 and f-CNT 1000 ex vivo (Fig. 6C, Additional file 1: Fig. S8B). The concentration of both gliotransmitters significantly increased in cerebellar slices incubated with f-CNTs. Then, we further used 5 µM tetrodotoxin (TTX), a drug that inhibits neuronal activity by blocking voltage-gated sodium channels in the neurons, to confirm that the increments of GABA and glutamate were derived from astrocytes. These molecules also increased even in TTX-treated cerebellar slices, suggesting that the increase of GABA and glutamate in brain slices was obtained from astrocytes (Fig. 6D, Additional file 1: Fig. S8). These results implying that f-CNTs upregulate GABA and glutamate in astrocytes and enhance the probability of interaction with neurons by releasing gliotransmitters. Synaptic clearance is astrocytes' major function that interacts with neurons and regulates homeostasis [19]. Therefore, we performed glutamate uptake assay in astrocytes on CNT platforms. The results showed that the uptake ratio of astrocytes on CNT 100 was improved, whereas cells on CNT 1000 showed no difference (Additional file 1: Fig. S8). Since the hydrophilicity of CNT 100 is superior to that of CNT 1000, there is a possibility that astrocytes stably perform their original function of clearing neurotransmitters (Fig. 2B). These results suggest that the characteristics of f-CNTs and CNT platforms can regulate astrocytes functions such as gliotransmission and synaptic clearance.

### CNT platforms enhance gliotransmission by increasing intracellular Ca^2+^ via TRPV1 channel

**Fig. 7:**
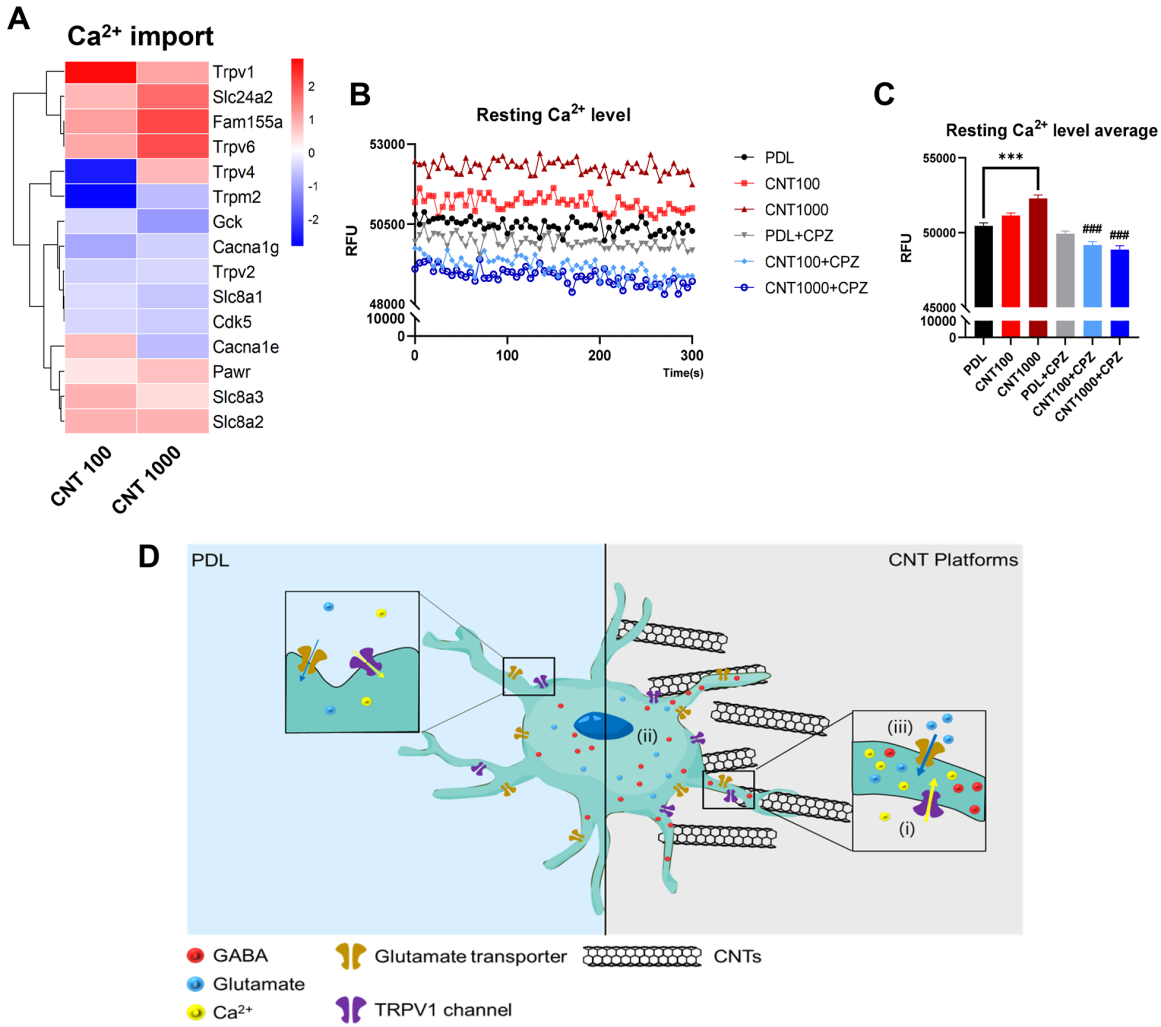
Intracellular Ca^2+^ level of astrocytes on MW-CNTs. **A** Heat map illustrating 3′ mRNA seq expression of Ca^2+^ import-associated genes of primary astrocytes on PDL and CNT platforms. Illustrating with reference to GO:0070509. **B** Resting calcium measurement traces and **C** average bar graph (n = 6 independent experiments). ###P < 0.0005 versus untreated astrocytes. **D** Schematic figure of astrocyte function on CNT platforms. (i) Astrocytic resting Ca^2+^ increased by upregulating TRPV1 expression. (ii) Due to increasing Ca^2+^ level, more gliotransmitters are secreted in astrocytes on functionalized CNTs and CNT platforms. (iii) Glutamate uptake was also improved in astrocytes on CNT platforms

Astrocyte functions are closely related to intracellular Ca^2+^ levels, essential for gliotransmission [12, 42]. We confirmed that the Ca^2+^ signaling pathway was significantly affected by CNT platforms using Pathview based on sequencing data (Tables 2 and 3) and selected genes related to Ca^2+^ import. The heatmap showed that Ca^2+^ import-related genes were upregulated in astrocytes on CNT platforms (Fig. 7A). Among the several genes, we focused on the *Trpv1*, which has the most significantly altered in astrocytes on CNT platforms. TRPV1, a mechanosensitive channel, is a Ca^2+^-permeable to modulate neuronal functions [43, 44]. We observed that the resting Ca^2+^ level in astrocytes was higher than on PDL using Ca^2+^ indicator (Fig. 7B, C). Moreover, we treated the astrocytes with Capsazepine (CPZ), a TRPV1 antagonist, which did not affect the cells on PDL but significantly reduced on CNT platforms (Fig. 7B, C). These results suggest that TRPV1 expression was increased in astrocytes on CNT platforms, which increases intracellular Ca^2+^ level for gliotransmission. Contrary to the increased resting Ca^2+^ in astrocytes on CNT 1000, it did not change significantly in astrocytes on CNT 100 (Fig. 7C). As CNT 1000 have better conductivity than CNT 100 (Fig. 3A), the enhanced resting Ca^2+^ level of astrocytes on CNT 1000 occurred due to the difference in the microenvironment recognized by cells. Moreover, the previous study has reported that CNT interacts with extracellular pores of TRPV1 [45]. These results suggest that CNT platforms may induce gliotransmission by upregulating intracellular gliotransmitters (Fig. 6), which is caused by increased Ca^2+^ level in astrocytes via TRPV1.

**Table 2:** Significant difference in Calcium signaling in Pathview analysis

CNT length	Time	p value	Stat mean	q value
100 nm	4 h	0.0104	2.3193	0.9597
1000 nm	4 h	0.0629	1.5341	0.9991
100 nm	24 h	0.0006	3.2377	0.027
1000 nm	24 h	0.0383	1.7756	0.2473

## Conclusions

This study shows that CNT platforms improve proliferation by inducing stable attachment of astrocytes. Interestingly, astrocyte functions related to gliotransmission via intracellular Ca^2+^ and synaptic clearance were enhanced by the electrical and nano-topographic properties of CNTs. In addition, gliotransmitters from astrocytes in cerebellum were regulated when functionalized CNTs were co-incubated ex vivo. Therefore, this study suggests that CNTs could be effective nanomaterials for therapeutic approach by using gliotransmitters from astrocytes and grafting cells on CNT platforms in the brain.

## Materials & methods

### Materials

Pristine CNT (Multi-walled carbon nanotubes, > 95%, 10–20 μm length, 15–20 nm outside diameter) were purchased from EMP (EM-Power Co., LTD, Korea). 3-aminopropyltriethoxysilane (APTES) and 1-ethyl-3-(3-dimethylaminopropyl) carbodiimide (EDC) were purchased from Sigma-Aldrich (USA). All supplementary chemicals were of analytical grades and used without further purification. Coverslips (12 mm, 30 mm diameter) were purchased from Marienfeld (Germany).

### Preparation of highly water-soluble functionalized CNTs of varying lengths

Chemical (Step1) and physical (Step2) functionalization of pristine CNT were carried out with the oxidative acid method and a homogenizer (MN400BF, Micronox Corp., South Korea).

*Step 1*: Initially, 2 g pristine CNT was dispersed in 100 mL 1:1 H_2_SO_4_/HNO_3_ solution and refluxed at 80 °C for 4 d. The mixture obtained was diluted in distilled water and filtered through a 0.4 μm Millipore polycarbonate filter membrane. The resulting carboxylated CNT powders (average length ~ 1000 nm) were then continuously washed till the filtrate pH reached 7 using distilled water.

*Step 2*: The residue from step1 (f-CNT 1000) was dispersed in distilled water and further cut using the high-pressure homogenizer (23,000 psi, 50 cycles). Representatively, a cycle of 50 repetitions reduced the CNT length from 1510.3 to 153.7 nm. The resulting CNT was ~ 100 nm long (f-CNT 100). Then, the physical functionalized CNT were freeze-dried.

### Immobilization of functionalized CNTs on the glass substrate for astrocyte platform

Before use, the glass substrates (coverslips) were ultrasonically cleaned in ethanol. The clean coverslips were immersed into 10% (w/v) APTES in distilled water containing HCl (pH = 3; adjusted with 2 N HCl) and incubated at 75 °C for 20 min. They were thoroughly rinsed in deionized water, ethanol, and acetone five times and dried at 60 °C. Five amine functionalized coverslips were soaked in 15 mL 0.01% (w/v) aqueous CNT solution containing 37.04 μM EDC and HCl (pH = 5; adjusted with 1 N HCl) by shaking at room temperature (RT) for 3 h. They were then rinsed with distilled water and ethanol and air-dried. The mono-layered CNT coating was confirmed using FE-SEM and AFM (Fig. 2E, F).

### Physicochemical and morphologic characterizations

The mean sizes of the carboxyl group functionalized CNTs were measured at RT using a Zeta sizer Nano ZS90 (Malvern, France) in an aqueous solution.

The CNT samples were analyzed qualitatively and quantitatively using FTIR (470 PLUS, JASCO, USA), TGA (TGA-1500 N, Sinco, Japan), and Raman spectroscopy (RAMAN, XploRA, Horiba, Japan).

L_O_, the in-plane crystallite size of CNT, was calculated from the RAMAN spectra. (Eq. 1)

1 $${\mathrm{L}}_{O} (\mathrm{nm}) = 2.4 \times {10}^{-10}{\uplambda }^{4} {\mathrm{I}}_{G}/{\mathrm{I}}_{D}$$

where λ is the laser wavelength, while I_*G*_ and I_*D*_ represent the integration intensity of the G and D band peak in the spectra, respectively.

The dispersion stabilities of functionalized CNTs were tested using a Turbiscane Lab (Leanontech, France) in phosphate buffered saline (PBS) solution at 37 ℃ for 1.5 h and measuring the backscattered light of a pulsed near-infrared light source of wavelength 880 nm.

The surface morphologies of CNT platforms were observed using FE-SEM (MIRA II LMH, Tescan, Czech Republic) and AFM (SPM-9700, Shimadzu, Japan). The resistance of CNT substrates was tested using Hiresta-UX MCP-HT800 (Nittoseiko analytech, Japan).

### Primary cerebellar astrocyte cultures

All animal experiments described below were performed in accordance with Dankook University Animal Experimentation Guidelines (approval number DKU-19–016, Cheonan, Korea). Cerebellum from P0 to P2 postnatal C57BL/6 mice was dissected free of adherent meninges, minced, dissociated into a single-cell suspension by pipetting, and cultured on 60 mm culture dish coated with 0.1 mg/mL poly-D-lysine (PDL; #354,210, Corning) in Dulbecco’s modified Eagle’s medium (DMEM, LM001-05, Welgene) supplemented with 25 mM glucose, 10% heat-inactivated horse serum (#26,050–088, Gibco), 10% heat-inactivated fetal bovine serum (FBS, #S001-01, Welgene), 2 mM glutamine, and 1% penicillin–streptomycin (#LS202-02, Welgene) at 37 °C in a humidified atmosphere containing 5% CO_2_. The cell debris and medium were removed after 3 days and fresh medium was added.

### Quant-seq mRNA 3′ sequencing analysis

#### RNA isolation

Total RNA was isolated using Trizol reagent (Invitrogen). RNA quality was assessed by Agilent 2100 bioanalyzer using the RNA 6000 Nano Chip (Agilent Technologies, Amstelveen, The Netherlands), and RNA quantification was performed using ND 2000 Spectrophotometer (Thermo Inc., DE, USA).

#### Library preparation and sequencing

For control and test RNAs, the library was constructed using Quant-seq 3′ mRNA Seq Library Prep Kit (Lexogen, Inc., Austria) according to the manufacturer’s instructions. Briefly, each total RNA was prepared, hybridized to an oligo dT primer containing an Illumina compatible sequence at its 5′ end, and reverse transcribed. After degradation of the RNA template, second strand synthesis was initiated by a random primer containing an Illumina compatible linker sequence at its 5′ end. The double stranded library was purified using magnetic beads to remove all reaction components. The library was amplified to add the complete adapter sequences required for cluster generation. The finished library is purified from PCR components. High throughput sequencing was performed as single end 75 sequencing using NextSeq 500 (Illumina, Inc., USA).

#### Data analysis

Quant-seq 3′ mRNA Seq reads were aligned using Bowtie2 (Langmead and Salzberg, 2012). Bowtie2 indices were generated from genome assembly sequence or the representative transcript sequences for aligning to the genome and transcriptome. The alignment file was used for assembling transcripts, estimating their abundances and detecting differential gene expression. Differentially expressed genes were determined based on the unique and multiple alignment count using coverage in Bedtools (Quinlan AR, 2010). The RC (Read Count) data were processed based on TMM + CPM normalization method using EdgeR within R (R development Core Team, 2020) using Bioconductor (Gentleman et al., 2004). Gene classification was based on searches in DAVID (http://david.abcc.ncifcrf.gov/) and Medline databases (http://www.ncbi.nlm.nih.gov/). Data mining and graphic visualization were performed using ExDEGA (Ebiogen Inc., Korea).

### Immunocytochemistry

Primary cerebellar astrocytes seeded on CNT platforms and PDL coverslips were fixed after 4 h and 24 h in 4% paraformaldehyde (PFA) for 15 min at RT. After fixation, the cells were washed thrice in 0.1 M PBS and incubated for 1 h at RT with blocking solution (0.3% Triton-X, 2% normal serum in 0.1 M PBS). The cells were then incubated with primary antibody diluted in blocking solution overnight at 4 °C on a shaker. The following primary antibodies were used: rabbit monoclonal anti-yes associated protein 1 (YAP, #14,074, Cell Signaling), mouse monoclonal anti-5′-Bromo-2-deoxyuridine (BrdU, #B2531, Sigma-Aldrich), chicken polyclonal anti-GFAP (#AB5541, Millipore Bioscience Research), guinea pig polyclonal anti-GABA (#AB175, Millipore Bioscience Research). F-actin was stained using Alexa Fluor™ 488-conjugated phalloidin (#A12379, Invitrogen™). After washing thrice with PBS, the cells were incubated with the corresponding secondary antibodies; Alexa Fluor™ 594-conjugated goat polyclonal anti-rabbit (#111-585-003, Jackson ImmunoResearch Inc.), Alexa Fluor™ 488-conjugated donkey polyclonal anti-chicken (#103-545-155, Jackson ImmunoResearch Inc.), and rhodamine (TRITC)-conjugated goat anti-guinea pig (#106-025-008, Jackson ImmunoResearch Inc.) for 90 min, followed by one rinse in PBS, and were then incubated with 4′,6-diamidino-2-phenylindole dihydrochloride (DAPI, #D9542; Sigma-Aldrich), followed by further washing in PBS. Then cells were then mounted in fluorescent mounting medium (#S3023, DAKO). A series of fluorescence images was obtained using confocal microscopy (Zeiss, LSM 700) and images were analyzed using ZEN 2010 imaging software. YAP fluorescence intensity was measured using imageJ and Excel program according to the following calculation:

$$YAP\, fluorescence\, intensity \left(i.u.\right)=cell\, intensity -(area\times mean\, background\, fluorescence)$$

### RNA extraction, cDNA synthesis, and real-time quantitative-PCR (qPCR)

First, primary cerebellar astrocytes were seeded on PDL and CNT platforms for 4 h and 24 h. Total RNA was extracted from cells using FavorPrep™ Tri-RNA Reagent (#FATRR 001, Favrogen Biotech Corp). Total RNA concentration was determined using a NanoDrop ND-1000 (Thermo-Fisher Scientific). The cDNA was then synthesized from 100 ng/µL total RNA using M-MLV Reverse Transcriptase (#28025013, Invitrogen™) according to manufacturer’s instructions. qPCR was performed on CFX Connect Real-Time PCR Detection System (Bio-Rad) with SYBR Green Realtime PCR Master Mix (#QPK-201, Toyobo) to detect *ITGAV*, *ITGB3*, and *GFAP* mRNA expression levels. The reference gene *HPRT* was used as control. Relative gene expression was normalized against *HPRT* expression and analyzed using BIO-RAD CFX Maestro 1.9 software (Bio-Rad). *ITGAV* and *ITGB3* primer sequences are listed in Table 3. The cDNA for analyzing *GFAP* gene expression was semi-quantified using commercially available primer sets (Bioneer). The entire system and all experiments were conducted in accordance with MIQE (Minimum Information for Publication of Quantitative Real Time PCR Experiments) guidelines.

**Table 3:** Primer sequence for qRT-PCR analysis

Target genes		Sequence (5' → 3')
*Hprt*	Forward	GCTGGTGAAAAGGACCTCT
	Reverse	CACAGGACTAGAACACCTGC
*ItgaV*	Forward	CAATTAGCAACACGGACTGC
	Reverse	CGTCACCATTGAAGTCTCCC
*Itgb3*	Forward	TTTGAGGAAGAACGAGCCAG
	Reverse	CCCGGTAGGTGATATTGGTG

### Measurement of cell viability and proliferation

Primary cerebellar astrocytes (2 × 10^5^ cells) were seeded for 1 d and the cytotoxicity of CNT platforms was compared to that of PDL-coated coverslips by incubating with Cell Counting Kit-8 (CCK-8; Enzo life science) solution for 2 h. The optical density (OD) was measured at 450 nm using the Epoch microplate reader (BIOTEK). Rates of cell cytotoxicity and proliferation rates were calculated from the following equation:

$$Cell\, viability={OD}_{sample}-{OD}_{blank}$$

where OD_blank_ was the OD of the medium alone.

### Protein extraction and western blot analysis

Primary cerebellar astrocytes were first seeded on PDL and CNT platforms for 24 h. Then, the proteins were extracted using RIPA buffer (50 mM Tris–HCl, 150 mM NaCl, 0.1% Triton-X 100, 0.1% sodium dodecyl sulfate (SDS), 5 mM EDTA) containing complete Tablets, Mini, EDTA-free protease inhibitor cocktail (#04 693 159 001, Roche) and centrifugation at 13000 rpm for 10 min. Then supernatant was collected, and protein concentration was determined using Pierce™ BCA Protein Assay Kit (#23,225, Thermo-Fisher Scientific). For western blotting, the cell lysates were denatured at 100 ℃ in 5 × sample buffer (60 mM Tris–HCl, 2% SDS, 25% glycerol, 5% β-merchaptoethanol, 0.1% bromophenol blue) for 5 min. Each sample (15 µg protein) was loaded on a 10% SDS–polyacrylamide gel, separated, and transferred to a Immobilon-P PVDF membrane (Millipore Bioscience Research). The membrane was blocked using blocking buffer (5% skim milk in Tris buffered saline-T (TBS-T), 100 mM Tris–HCl, 150 mM NaCl, 0.05% Tween-20) for 1 h at RT and then incubated with primary antibodies. The following antibodies were used: chicken polyclonal anti-GFAP (#AB5541, Millipore Bioscience Research) or mouse monoclonal anti-β-actin (#sc47778, Santa Cruz). After incubation at 4 ℃ overnight, the membranes were washed thrice using TBS-T and incubated with secondary antibodies; horseradish peroxidase (HRP)-conjugated goat polyclonal anti-rabbit (#A120-101P, Bethyl Laboratories. Inc) and anti-mouse (#A90-116P, Bethyl Laboratories. Inc) for 1 h at RT, and washed thrice with TBS-T. Then, the membranes were scanned and analyzed using the ChemiDoc XRS + imaging system (Bio-Rad) and ImageJ software according to manufacturer’s instructions. The primary antibodies were diluted in blocking buffer and secondary antibodies were diluted in TB S-T.

### Liquid chromatography/mass spectrometry

To measure the gliotransmitter level of astrocytes on PDL and CNT platforms, the cells were first cultured on each material-coated coverslips for 24 h to collect the lysates and soluble fractions. Agilent 6410B Triple Quadrupole liquid chromatography/mass spectrometry (LC/MS, Agilent Technologies, Wilmington, USA) equipped with an ESI source was employed for the analysis. GABA and glutamate were used as reference standards. 100 mg sample was mixed with 900 µL methanol and centrifuged at 10,000 rpm for 10 min. Then, 5 µL processed sample was injected into the HPLC system (1200 Series LC, Agilent Technologies) fitted with Phenomenex Synergi Hydro-RP 4 µm 80 Å 150 × 2 mm column, maintained at 30 °C. ESI was operating at + 3000 V and 380 °C source temperature. Capillary voltage, cone voltage, and source offset were set at 3 kV, 30 kV and 30 V respectively. The gas flow of desolvation and the cone was set at 650 and 150 L/h, respectively, with a nebulizer pressure of 15 bar. A mobile phase comprising 0.1% formic acid in distilled water (Buffer A) and 0.1% formic acid in acetonitrile (Buffer B) was used to separate the analytes and pumped into the ESI chamber at 0.5 mL/min flow rate for 20 min. The fragmentor voltage and collision voltage was set at 70 V. The ions were detected in the multiple-reaction monitoring mode (MRM) by monitoring the m/z transition pairs 104 → 87 (GABA) and 148 → 84 (glutamate). Data acquisition was performed with the MassHunter Software (Version B.04.00).

### Enzyme-linked immunosorbent assay

To measure the GABA level of astrocytes on PDL and CNT platforms, cells were first cultured on different material-coated coverslips for 24 h to collect the lysates and soluble fractions following protein extraction protocol. The endogenous and secreted gliotransmitter GABA were then measured by commercial GABA ELISA kit (#BA-E 2500, LDN) following the manufacturer’s guidelines. To quantity the GABA level in cerebellar slices, artificial cerebrospinal fluid (aCSF; 130 mM NaCl, 24 mM NaHCO_3_, 1.25 mM NaH_2_PO_4_, 3.5 mM KCl, 1.5 mM CaCl_2_, 1.5 mM MgCl_2_, and 10 mM D ( +) ‐glucose; pH 7.4) and brain slices were collected after incubation with functionalized CNTs. Then, the GABA levels in the collected samples were measured by same ELISA kit following the manufacturer’s instructions.

### Glutamate assay

To measure the glutamate level of astrocytes, the cells were cultured on PDL and CNT platforms for 24 h to collect the lysates and supernatants following protein extraction protocol. The secreted gliotransmitter glutamate was then measured using the commercial Glutamate Assay Kit (#ab83389, Abcam) following the manufacturer’s guidelines. To quantity the glutamate level of brain slices, aCSF and cerebellar slices were collected after incubation with functionalized CNTs. Then, the glutamate levels in the collected samples were measured by same kit following the manufacturer’s instructions. For glutamate uptake experiments, the astrocytes were incubated for 30 min in Hank’s balanced salt solution (HBSS) buffer without Ca^2+^ and Mg^2+^ (#14,185,052, Gibco), then for 3 h in HBSS with Ca^2+^ and Mg^2+^ (#14,025,092, Gibco) containing 100 μM glutamate. Simultaneously, equal volumes of HBSS with Ca^2+^ and Mg^2+^ containing 100 μM glutamate were incubated in empty wells for determining the percentage of glutamate uptake. The medium of each well was collected after 2 h and analyzed using a Glutamate Assay Kit (#ab83389, Abcam) according to manufacturer’s instructions. HBSS without Ca^2+^ and Mg^2+^ was also used as the negative control.

### Brain slice

P49–56 C57BL/6 adult male mice were deeply anesthetized with halothane. After decapitation, the brain was quickly excised from the skull, submerged in ice-cold artificial cerebrospinal fluid (aCSF). and gassed with 5% CO_2_ balanced O_2_. After trimming the cerebellum, 300 µm sagittal slices were cut using a vibratome and transferred to extracellular aCSF solution. Then, the cerebellar slices were incubated with aCSF and functionalized CNTs (10 µg/mL) for 3 h before collecting slices and aCSF. After incubation, the slices and solvents were used for GABA ELISA and glutamate assay.

### Measurement of resting intracellular Ca^2+^

To measure the intracellular Ca^2+^ level in astrocytes, the cells were seeded on PDL and CNT platforms for 24 h. Then, the cells were washed with HEPES buffer (200 mM HEPES, 2 mM MgCl_2_, 2 mM CaCl_2_, 5.5 mM D-glucose, 21 mM sucrose). After washing with HEPES buffer, intracellular Ca^2+^ was labeled by 1 μM Fluo-4, AM, cell permeant (#F14201, Invitrogen) in HEPES buffer following manufacturer’s instructions. Then, fluorescence endpoint was measured and analyzed using Synergy HTX Multi-Mode Reader (BIOTEK) and Gen5 (Ver. 2.09.2).

### Statistical analysis

Off-line analysis was carried out using GraphPad Prism 9.3.0 (463) and Excel software. The significance of data for comparison was assessed by Student’s two-tailed unpaired *t*-test and one-way ANOVA. The general data distribution was assumed to be normal, but this was not formally tested. Data are presented as mean ± SEM (standard error of the mean). Levels of statistical significance are as follows: *(p < 0.05), **(p < 0.005), ***(p < 0.0005), and ****(p < 0.00005). Quant-seq data was analyzed using Pathview (ver.1.32.0) and pheatmap (ver.1.0.12) in R (ver.4.1.0).

## Supplementary Information

**Additional file 1: Figure S1.** Description of materials and methods; colloidal stability of f-CNT samples by cycles. **Figure S2.** Characterization of FT-IR and TGA of pristine CNT, f-CNT 100 and f-CNT 1000. **Figure S3.** XPS full spectrum of the substrate before, during, and after functionalized CNT and contact angle of 12, 30 mm glass. **Figure S4.** Photograph of 12- and 30-mm glass substrates before and after f-CNT coating. **Figure S5.** Additional AFM images of glass substrates coated with or without CNT. **Figure S6.** Low magnification FE-SEM images of 12- and 30-mm glass substrates. **Figure S7.** Yap expression of astrocyte on CNT platforms. **Figures S8.** Glutamate uptake of astrocytes on CNT platforms.

## Data Availability

The datasets used and/or analyzed during the current study are available from the corresponding author on reasonable request.

## Abbreviations

aCSF: Artificial cerebrospinal fluid
AFM: Atomic field microscopy
APTES: 3-aminopropyltriethoxysilane
BDNF: Brain-derived neurotrophic factor
BrdU: Bromodeoxyuridine
CNS: Central nervous system
CNT: Carbon nanotube
ECM: Extracellular matrix
GABA: γ-Aminobutyric acid
GDNF: Glial cell-derived neurotrophic factor
GFAP: Glial fibrillary acidic protein
f-CNT 100: Functionalized 100 nm CNT-coated glass substrate
f-CNT 1000: Functionalized 1000 nm CNT-coated glass substrate
FE-SEM: Field emission scanning electron microscopy
FTIR: Fourier-transform infrared spectroscopy
ID/IG: Intensity of D band over intensity of G band
ItgaV: Integrin αV
Itgb3: Integrin β3
LO: In-plane crystalline size
MW-CNT: Multi-wall carbon nanotube
PDL: Poly-D-lysine
RT: Room temperature
RAMAN: RAMAN spectroscopy
TGA: Thermogravimetric analysis
TRPV1: Transient receptor potential vanilloid type 1
TTX: Tetrodotoxin
XPS: X-ray photoelectron spectroscopy
YAP: Yes-associated protein
