# Supplementary material for: Improved gliotransmission by increasing intracellular Ca2+ via TRPV1 on multi-walled carbon nanotube platforms
Source: J Nanobiotechnology. 2022 Aug 11;20:367. doi: 10.1186/s12951-022-01551-1 (PMC9367080; doi:10.1186/s12951-022-01551-1)
Supplement: Supplementary file 1 — Additional file 1: Figure S1. Description of materials and methods; colloidal stability of f-CNT samples by cycles. Figure S2. Characterization of FT-IR and TGA of pristine CNT, f-CNT 100 and f-CNT 1000. Figure S3. XPS full spectrum of the substrate before, during, and after functionalized CNT and contact angle of 12, 30 mm glass. Figure S4. Photograph of 12- and 30-mm glass substrates before and after f-CNT coating. Figure S5. Additional AFM images of glass substrates coated with or without CNT. Figure S6. Low magnification FE-SEM images of 12- and 30-mm glass substrates. Figure S7. Yap expression of astrocyte on CNT platforms. Figures S8. Glutamate uptake of astrocytes on CNT platforms. [file 12951_2022_1551_MOESM1_ESM.docx]

Supporting information

Improved gliotransmission by increasing intracellular Ca^2+^ via TRPV1 on multi-walled carbon nanotube platforms

Won-Seok Lee^1, 3, 6‡^, Ji-Hye Kang^2, 3‡^, Jung-Hwan Lee^2, 3, 4, 5, 6^ Yoo Sung Kim^1^, Jongmin Joseph Kim^1^, Han-Sem Kim^3^, Ueon Sang Shin^2, 3*^ and Bo-Eun Yoon^1,3, 6*^.

1) Department of Molecular biology, Dankook University, Cheonan 31116, Republic of Korea.

2) Department of Nanobiomedical Science, BK21 FOUR NBM Global Research Center for Regenerative Medicine, Dankook University, Cheonan 31116, Republic of Korea.

3) Institute of Tissue Regeneration Engineering (ITREN), Dankook University, Cheonan 31116, Republic of Korea.

4) Department of Biomaterials Science, College of Dentistry, Dankook University, Cheonan 31116, Republic of Korea.

5) UCL Eastman-Korea Dental Medicine Innovation Centre, Dankook University, Cheonan 31116, Republic of Korea.

6) Mechanobiology Dental Medicine Research Center, Cheonan 31116, Republic of Korea

**Supplementary figure**


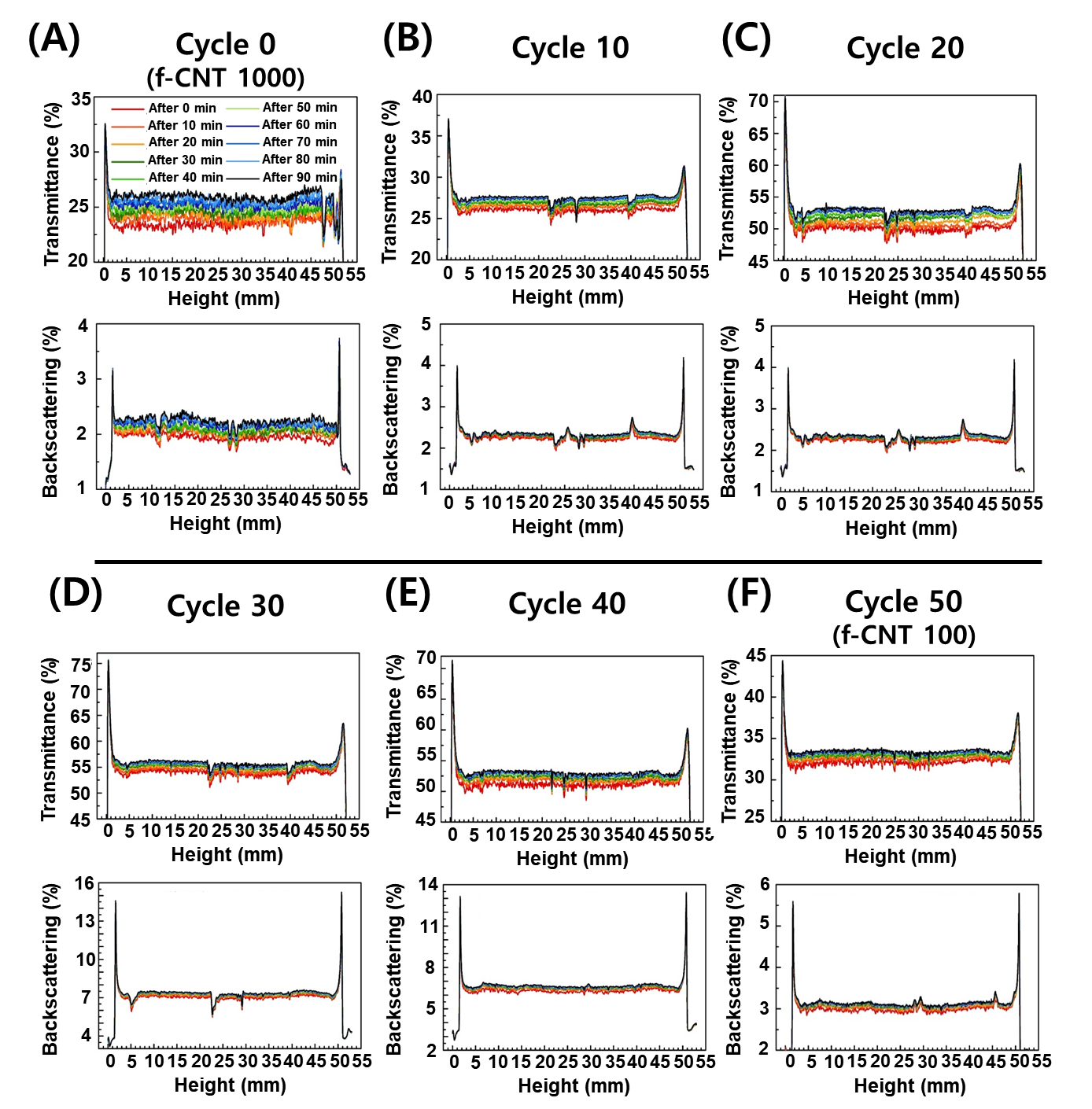


**Fig. S1.** **Colloidal stability of physically functionalized carbon nanotube (CNT) samples by cycles**. (A) 0, (B) 10, (C) 20, (D) 30, (E) 40, and (F) 50 cycles.


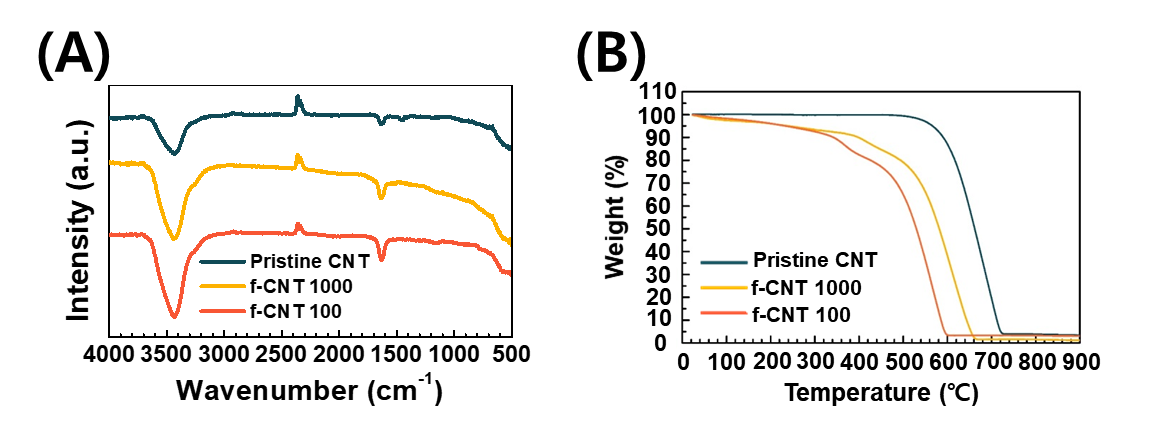


**Fig. S2.** Characteristic analysis of pristine carbon nanotube (CNT), functionalized 100 nm CNT (f-CNT 100), and functionalized 1000 nm CNT (f-CNT 1000). (A) Fourier-transform infrared spectroscopy (FTIR) and (B) thermogravimetric analysis (TGA) spectra.


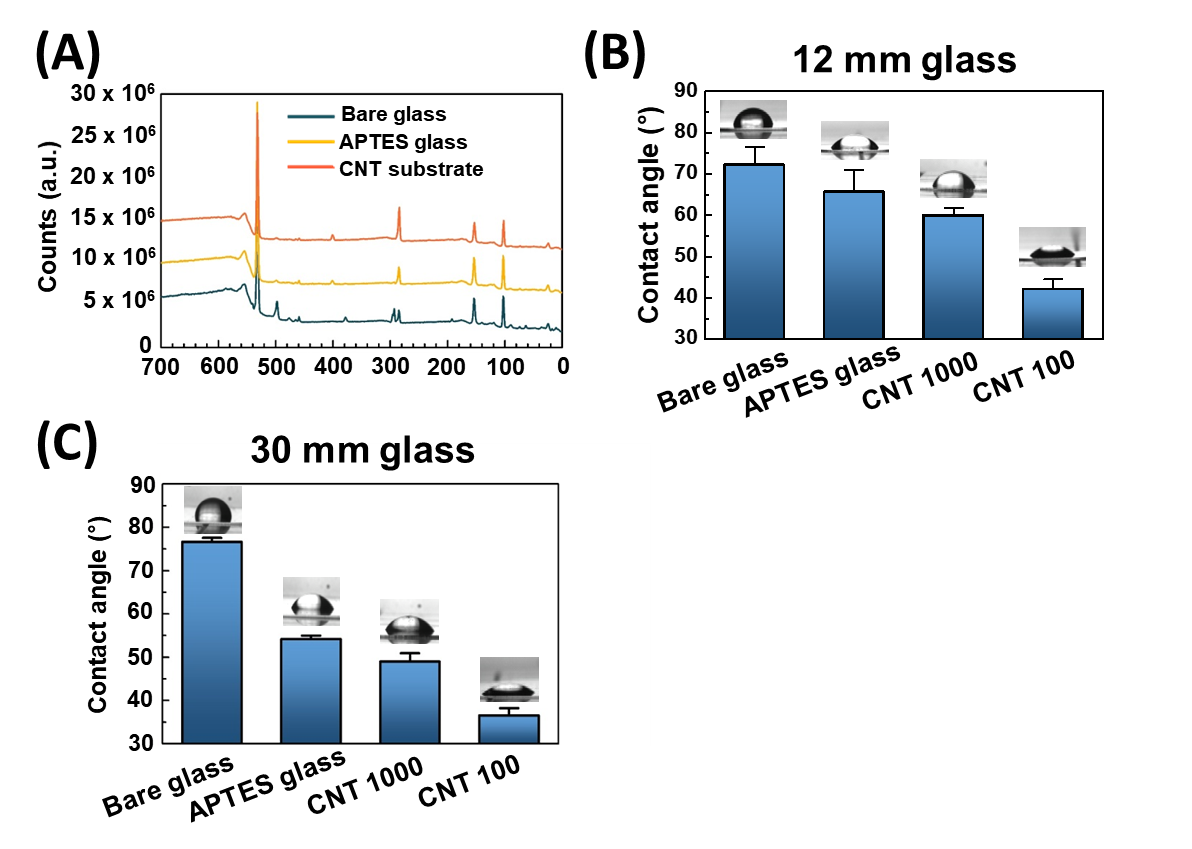


**Fig. S3.** Characterization data for each coating process (A) XPS full spectrum of the substrate before, during, and after the functionalized carbon nanotube (CNT) coating. Contact angle of (B) 12 and (C) 30 mm glass.


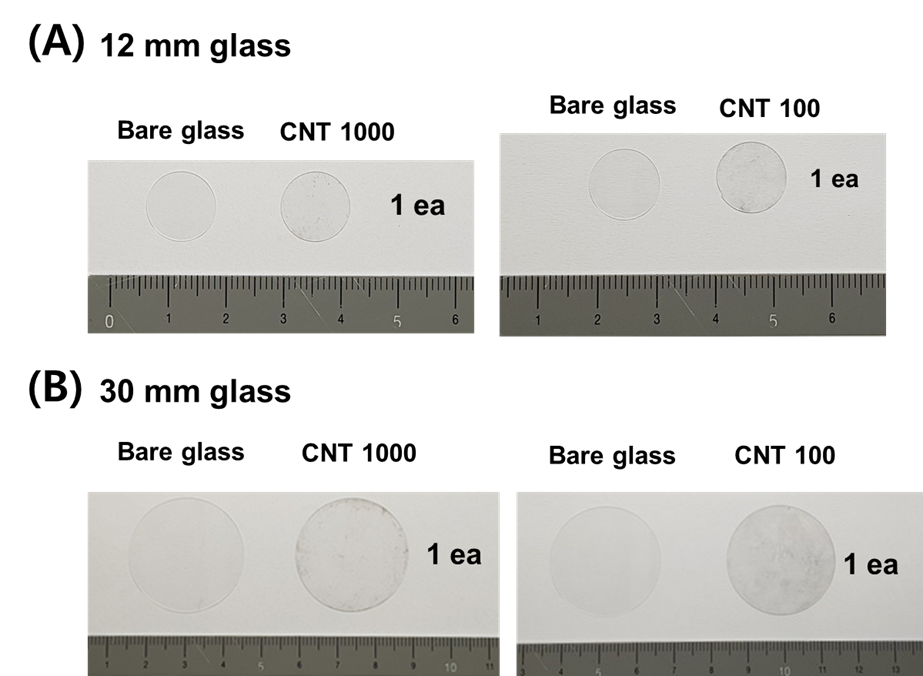


**Fig. S4.** Photograph of 12- and 30-mm glass substrates before and after coating with functionalized carbon nanotubes (CNTs) (CNT 100: 100 nm carbon nanotube, CNT 1000: 1000 nm carbon nanotube).


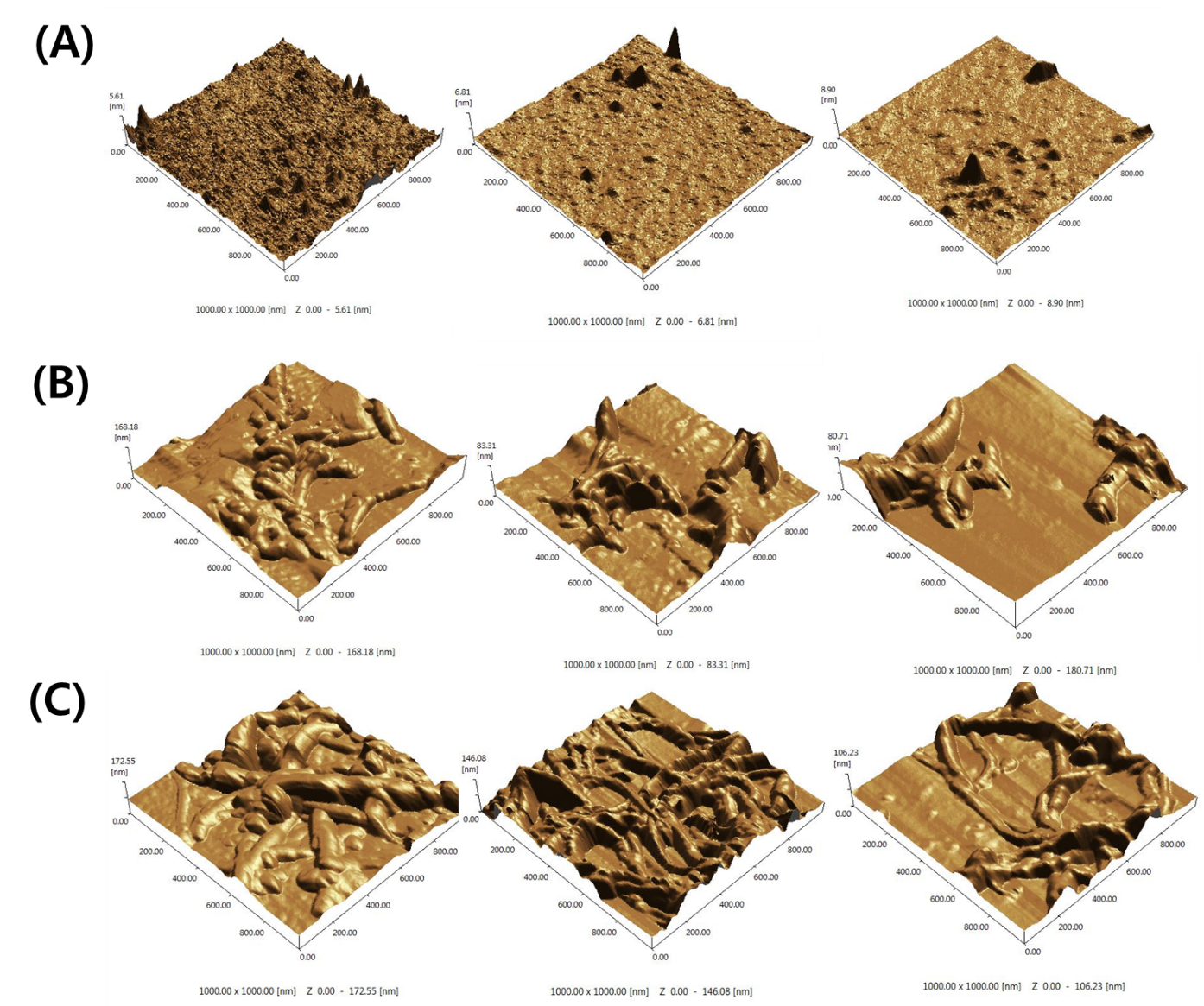


**Fig. S5.** Additional Atomic force microscopy (AFM) images of glass substrates coated with or without carbon nanotube (CNT). (A) poly-D-lysine (PDL)-coated glass, (B) 100 nm CNT-coated glass (CNT 100), and (C) 1000 nm CNT-coated glass (CNT 1000).


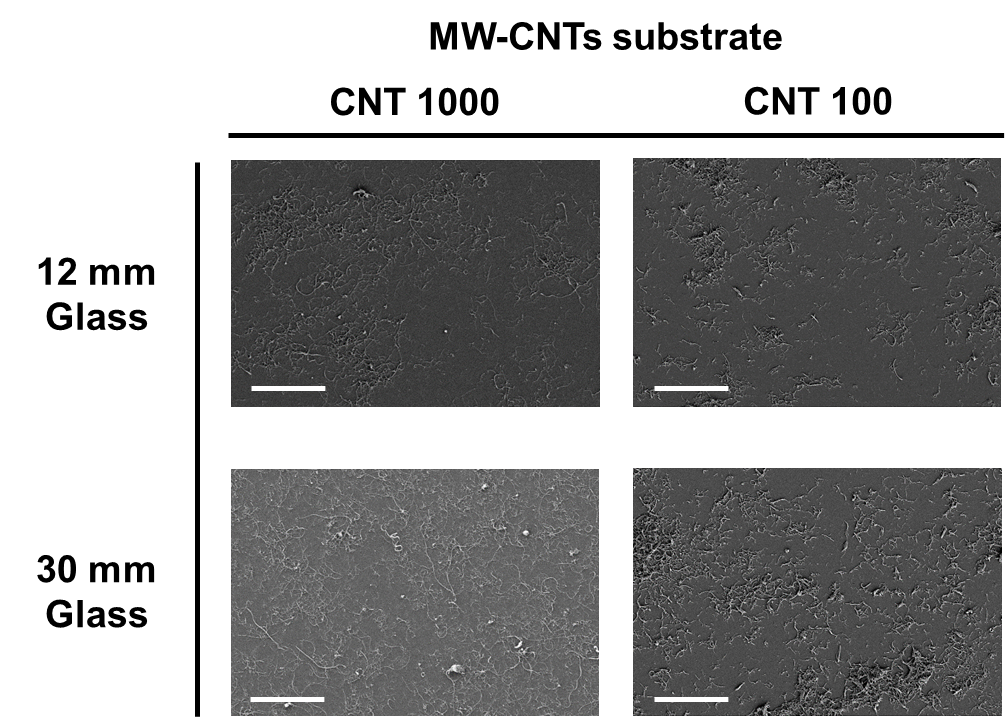


**Fig. S6.** Low magnification field emission scanning electron microscopy (FE-SEM) images of 12- and 30-mm glass substrates coated with functionalized carbon nanotube (CNT 100: 100 nm CNT-coated glass, CNT 1000: 100 nm CNT-coated glass; Scale bar: 5 μm)


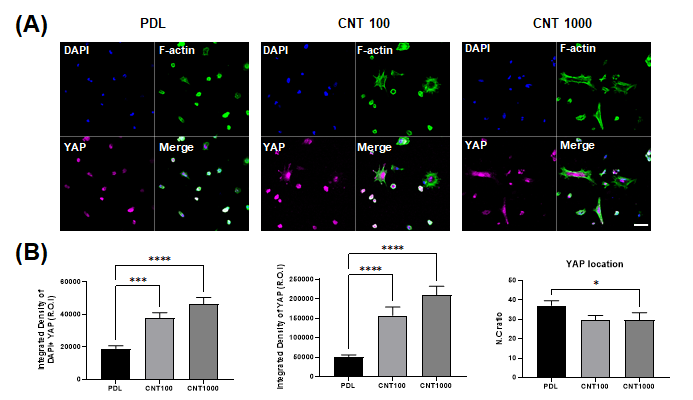


**Fig. S7. YAP expression of astrocytes on MW-CNTs.** (A) Carbon nanotube (CNT)-coated platforms increase YAP immunoreactivity than poly-D-lysine (PDL). (B) Data are presented as the mean (n=30 cells). *P<0.05, **P<0.005, ***P<0.0005, and ****P<0.00005 versus PDL.

**(A)**

**(B)**

**Fig. S8 Glutamate of astrocytes on CNT platforms and cerebellar slices incubated with f-CNTs.** (A) Intracellular glutamate level of primary cerebellar astrocytes was measured using the glutamate enzyme assay kit. CNT platforms increased the amount of extracellular glutamate, and there was no difference in the total glutamate amount. Data are presented as the mean ± SEM (n=4). *P<0.05, **P<0.005, and ***P<0.0005 versus the PDL group. (B) Graphs show that functionalized CNTs increase glutamate levels in cerebellar astrocytes after neurotoxin treatment (TTX). Data presented as the mean ± SEM (n=4 individual mice).

**Fig. S9. Glutamate uptake assay of astrocytes on carbon nanotube (CNT) platforms.** Glutamate uptake enhanced in cerebellar astrocytes on 100 nm CNT-coated platform (CNT 100). Data are presented as the mean ± SEM (n=3 independent experiments). Uptake ratio was measured by optical density. *P<0.05 versus PDL.
